# Oxidative Stress and Cardiovascular Risk Factors: The Coronary Artery Risk Development in Young Adults (CARDIA) Study

**DOI:** 10.3390/antiox12030555

**Published:** 2023-02-23

**Authors:** Amir S. Heravi, Di Zhao, Erin D. Michos, Henrique Doria De Vasconcellos, Bharath Ambale-Venkatesh, Donald Lloyd-Jones, Pamela J. Schreiner, Jared P. Reis, James M. Shikany, Cora E. Lewis, Chiadi E. Ndumele, Eliseo Guallar, Pamela Ouyang, Ron C. Hoogeveen, Joao A. C. Lima, Wendy S. Post, Dhananjay Vaidya

**Affiliations:** 1 School of Medicine, Johns Hopkins University, Baltimore, MD 21287, USA; 2 Bloomberg School of Public Health, Johns Hopkins University, Baltimore, MD 21287, USA; 3 Feinberg School of Medicine, Northwestern University, Chicago, IL 60208, USA; 4 School of Public Health, University of Minnesota, Minneapolis, MN 55455, USA; 5 National Heart, Lung, and Blood Institute, Bethesda, MD 20892, USA; 6 School of Medicine, University of Alabama at Birmingham, Birmingham, AL 35294, USA; 7 School of Public Health, University of Alabama at Birmingham, Birmingham, AL 35294, USA; 8 Baylor College of Medicine, Houston, TX 77030, USA

**Keywords:** 2,3-dinor-8-isoprostane, 8-isoprostane, cardiovascular disease risk factors, oxidative stress, urinary isoprostanes

## Abstract

Introduction—Oxidative stress is linked to cardiovascular diseases (CVD) and is suggested to vary by sex. However, few population-level studies have explored these associations and the majority comprise populations with advanced CVD. We assessed urinary isoprostane concentrations, a standard measure of oxidative stress, in a relatively young and healthy cohort, hypothesizing that higher oxidative stress is associated with an adverse cardiometabolic profile and female sex. Methods—Oxidative stress was measured in 475 women and 266 men, aged 48–55 years, from the Coronary Artery Risk Development in Young Adults (CARDIA) study using urinary 8-isoprostane (IsoP) and 2,3-dinor-8-isoprostane (IsoP-M). Multivariable-adjusted regression was used to evaluate cross-sectional associations. As secondary analysis, previously measured plasma F2-isoprostanes (plasma IsoP) from another CARDIA subset was similarly analyzed. Results—Mean (SD) ages for men and women were 52.1(2.3) and 52.2(2.2) years, respectively (*p* = 0.46), and 39% of the participants self-identified as Black (vs. White). Before adjustments, female sex was associated with higher median urinary IsoP (880 vs. 704 ng/g creatinine in men; *p* < 0.01) and IsoP m (1675 vs. 1284 ng/g creatinine in men; *p* < 0.01). Higher body mass index (BMI), high-density cholesterol (HDL-C), and triglycerides, current smoking, and less physical activity were associated with higher oxidative stress. Diabetes was not associated with urinary IsoP but was associated with lower IsoP m and plasma IsoP. Higher serum creatinine showed diverging associations with higher plasma and lower urinary isoprostane concentrations. Conclusions—Different isoprostane entities exhibit varying association patterns with CVD risk factors, and therefore are complementary, rather than interchangeable, in assessment of oxidative stress. Still, consistently higher isoprostanes among women, smokers, less active persons, and those with higher BMI and plasma triglycerides could reflect higher oxidative stress among these groups. While urinary isoprostanes are indexed to urinary creatinine due to variations in concentration, caution should be exercised when comparing groups with differing serum creatinine.

## 1. Introduction

Oxidative stress—defined as perturbed balance with excess reactive oxygen species (ROS) vis-a-vis the body’s antioxidant defense system [1]—has been linked to the development of many cardiovascular diseases (CVD), including oxidation of lipoproteins [2], atherosclerosis [3] and heart failure [4]. Connections between oxidative stress and CVD risk factors such as diabetes [5], tobacco use [6], and endothelial dysfunction [7] have also been suggested. Furthermore, some suggest sex disparities in CVD, especially after menopause, may be partially attributable to sex differences in oxidative stress as the result of loss of potential protective effects of sex hormones on modulation of oxidative stress [8,9,10]. On the other hand, higher oxidative stress in premenopausal women compared with men is also reported [11]. Thus, the exact role of oxidative stress in the pathophysiology of CVD and whether true sex differences in oxidative stress exist remain equivocal [12,13].

Isoprostanes are prostaglandin-like byproducts of arachidonic acid peroxidation induced by ROS and are considered a standard marker for in vivo oxidative stress [14,15]. Isoprostanes are both mediators and indicators of oxidative stress and a measure of the redox status of the internal milieu across many human diseases [16]. Yet, few epidemiologic studies have characterized the relationship between CVD risk factors and isoprostanes. Moreover, the majority of existing studies were performed in populations with advanced CVD, rarely offered parallel assessments of different isoprostane metabolites or compared associations in plasma vs. urinary isoprostanes [17].

We measured urinary concentrations of 8-isoprostane (urinary IsoP) and its metabolite 2,3-dinor-8-isoprostane (urinary IsoP-M) in a subset of participants in a middle-aged and relatively healthy population-based cohort, and reanalyzed previously measured plasma F2-isoprostanes (plasma IsoP). We postulated that CVD risk factors, including behavioral risk factors such as smoking and physical activity, metabolic conditions such as diabetes, hypertension and adverse lipid profile and female sex would be independently associated with greater oxidative stress, as measured by higher urinary isoprostanes.

## 2. Materials and Methods

### 2.1. Study Population

Coronary Artery Risk Development in Young Adults (CARDIA) is a community-based, multicenter, observational, longitudinal cohort study sponsored by the National Heart, Lung, and Blood Institute. CARDIA enrolled 5115 women and men between 18 and 30 years of age, free from CVD, in 1985-86 from 4 centers (Birmingham, AL; Chicago, IL; Minneapolis, MN; and Oakland, CA, USA) [18]. The institutional review boards of all participating study sites approve the study annually, and all participants provide written informed consent.

**Figure 1:**
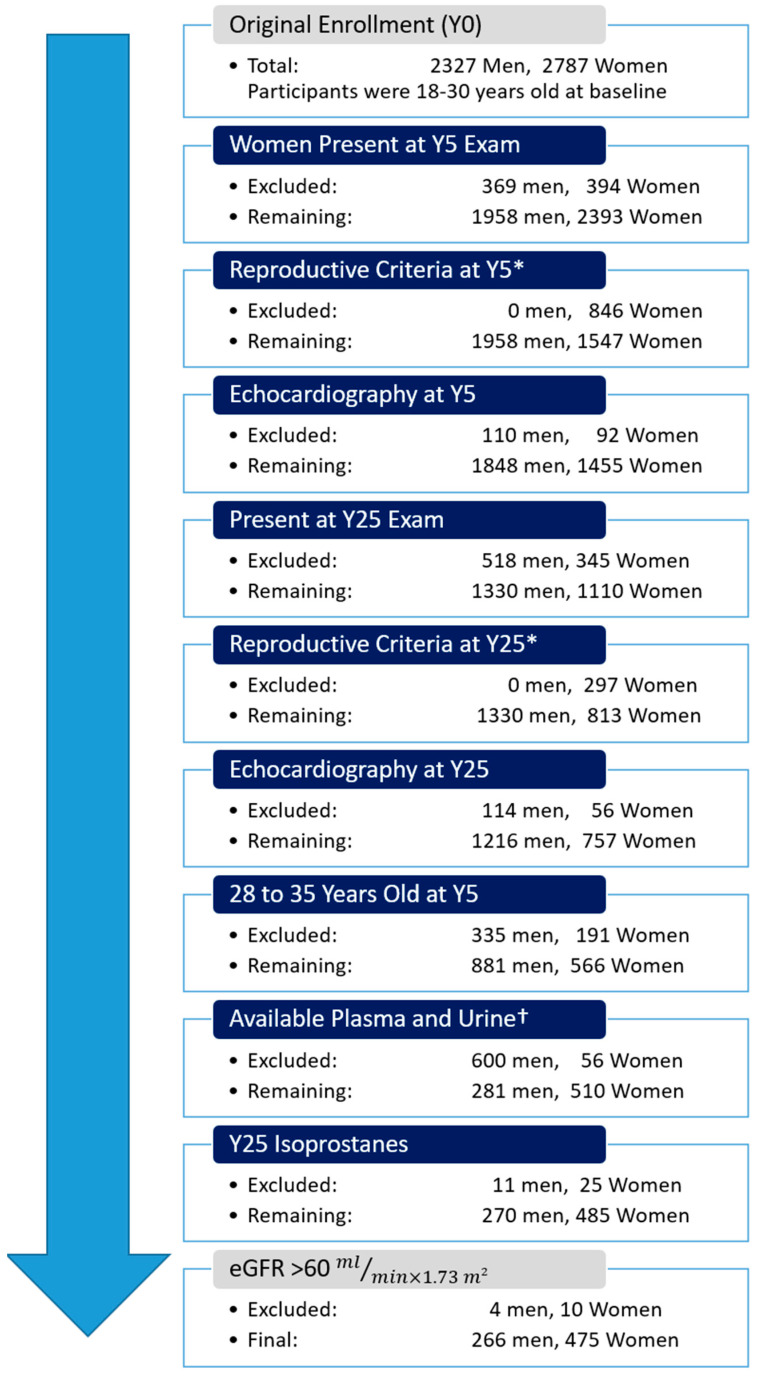
Inclusion/exclusion flow. * Reproductive criteria: known menopausal status and absence of pregnancy, breastfeeding or exogenous sex hormone use. † at both Y5 and Y25.

The present study is a cross-sectional analysis of CARDIA participants for whom urinary isoprostane concentrations were measured at the year 25 (Y25) follow-up exam (2010–2011) as part of an ancillary study. Complete inclusion and exclusion criteria for the ancillary study, for which the primary goal was to assess changes in cardiac structure and function during the menopausal transition, are listed in Figure 1. For this analysis, the study population comprised 48- to 55-year-old CARDIA participants who satisfied criteria for availability of questionnaire data, cardiac imaging, and biospecimens in line with the objectives of the parent ancillary study. Participants with estimated glomerular filtration rate (eGFR) <60 mL/min/1.73 m^2^ using the Modification of Diet in Renal Disease (MDRD) equation [19] were excluded to avoid interpretability issues for urine-based assays in the setting of impaired renal function. Based on the above criteria, 475 women and 266 men were included (Figure 1).

Plasma-free (non-esterified) IsoP concentrations from the year 15 (Y15) exam were available from another prior CARDIA ancillary study [20] which comprised 2999 participants at the Y15 exam with available computed tomography coronary artery calcium score. We used plasma IsoP measurements and their concurrent exposure variables for a secondary analysis, similar to that of the urinary markers.

### 2.2. Measurement of Exposure Variables

Assessment of exposure variables was performed for each visit using standardized questionnaires, physical exam, and laboratory measures, as described previously [18]. Body mass index (BMI) was calculated as weight (kg) divided by height (m) squared. All participants were asked to fast for 12 h before each clinic visit. Blood pressure was measured using the Omron device (Omron Healthcare Inc., Lake Forest, IL, USA) at Y25. Plasma total cholesterol, high-density lipoprotein cholesterol (HDL-C) and triglyceride concentrations were measured using enzymatic methods. Physical activity was assessed with the CARDIA physical activity questionnaire accounting for the frequency and intensity of physical activity (the metabolic equivalent of task for each exercise category multiplied by the sum of months of infrequent participation plus 3 times months of frequent participation in the prior year) and reported as “exercise units” (EU) [21]. Diabetes was defined as the presence of any of measured fasting glucose ≥126 mg/dL or use of glucose-lowering medications (in any previous exams), 2 h post-load glucose ≥200 mg/dL (during a 75 g oral glucose tolerance test at years 10, 20, and 25) or an HbA1c ≥6.5% (at years 20 and 25).

### 2.3. Isoprostane Measurements

Urinary IsoP and IsoP m were measured at the Atherosclerosis Clinical Research Laboratory at Baylor College of Medicine (Houston, TX, USA) using untimed urine samples typically collected midmorning after nocturnal fasting. Samples underwent sequential washing with solvent mixtures on mixed anion solid phase exchange columns for subsequent measurement of isoprostanes via gas chromatography-mass spectrometry (GC-MS) with negative chemical ionization [22]. Assay variance was assessed using two pools of quality control samples for each isoprostane entity and yielded intra-assay covariance of variance (CV) of 5–7% (Supplementary Table S1). To account for variations in urine concentration, each isoprostane measurement was indexed to urinary creatinine measured using the Jaffe rate method [23]. Plasma IsoP measurements were obtained from samples frozen promptly after collection, shipped overnight, and kept frozen at −80 °C until undergoing GC-MS within 1 year of collection. This process was reported to result in no ex vivo production or disintegration of isoprostanes. An internal standard was added to the samples to quantify assay isoprostane recovery and the analytical variation within 3 control pools was 10% or less [20]. Plasma IsoP measures total F2-isoprostanes, a composite of isomers among which 8-isoprostane is the best studied [16].

### 2.4. Statistical Analysis

Population characteristics were reported as mean ± standard deviation, median [interquartile range] or number (percentage) as appropriate, and group differences were tested using Student’s t-, rank sum or χ2 tests, respectively. Creatinine indexed urinary IsoP and IsoP-M, plasma IsoP, and plasma triglycerides were log-transformed due to right-skewed distribution. Cross-sectional associations between urinary isoprostane concentrations (as dependent variables in separate models) and CVD risk factors (independent variable) were explored using multivariable-adjusted linear regression. The models were progressively adjusted (except if a variable was already included as variable of interest, e.g., when evaluating BMI, Model 1 adjusts for age, race, sex, college attainment and study field center, since BMI itself is automatically included as an independent variable): Model 1 adjusts for age, race, sex, college attainment, BMI and study field center. Model 2 includes covariates from model 1 plus serum creatinine. For each CVD risk factor except smoking-related variables, Model 3 introduces additional adjustments for smoking status (current, former, or never), and cumulative pack years. Model 4 (our primary model) includes covariates in Model 3 plus diabetes, use of medication for hypertension, systolic blood pressure, use of lipid-lowering medication, total cholesterol, HDL-C, fasting triglyceride concentration (log transformed) and physical activity. In linear regression of tobacco use variables, smoking status and cumulative pack years were not mutually adjusted (Model 3 was removed), as the two were highly correlated (data not shown).

Since log-transformed isoprostane concentrations were used, the model results are presented as exponentiated beta-coefficients reflecting the association of each risk factor with isoprostane concentrations as a ratio (ratio > 1 indicates a positive and <1 indicates an inverse relationship). Two-sided *p*-values < 0.05 were considered statistically significant. All analyses were performed on Stata version 15.1 (StataCorp LP, College Station, TX, USA).

### 2.5. Sensitivity Analysis

The analyses were repeated after replacing the diabetes variable with Homeostatic Model Assessment of Insulin Resistance (HOMA-IR), fasting glucose, diabetes medication use, or hemoglobin A1c to assess robustness of the models. Separately, indexing by urinary creatinine was replaced by adjustment for this factor as an independent variable in each regression model [24]. In urinary isoprostane models, potential interactions between race and sex with each of the CVD risk factors of interest were explored and interpreted after application of Bonferroni correction. In plasma isoprostane models, analysis was repeated within the subgroup who also had Y25 urine isoprostanes.

## 3. Results

### 3.1. Population Characteristics and Urinary Assays

Characteristics at Y25 are shown in Table 1 (Y15 in Supplementary Table S2). Women and men were similar in age (mean 52.2 vs. 52.1 years, respectively), proportion identifying as Black (38.5% vs. 39.8%) and current smoking status (13.4% vs. 14.0%). Compared with men, women reported less physical activity and had lower systolic blood pressure, fasting triglyceride and glucose concentrations, but higher total and HDL cholesterol concentrations. Women were less likely to use medications for hypertension, less likely to have diabetes and had lower urinary and serum creatinine concentrations compared with men. Median concentrations of both urinary isoprostanes (indexed to urinary creatinine) were higher in women than men (25% higher for IsoP and 30% higher for IsoP-M).

### 3.2. Urinary IsoP vs. Cardiovascular Risk Factors

**Figure 2:**
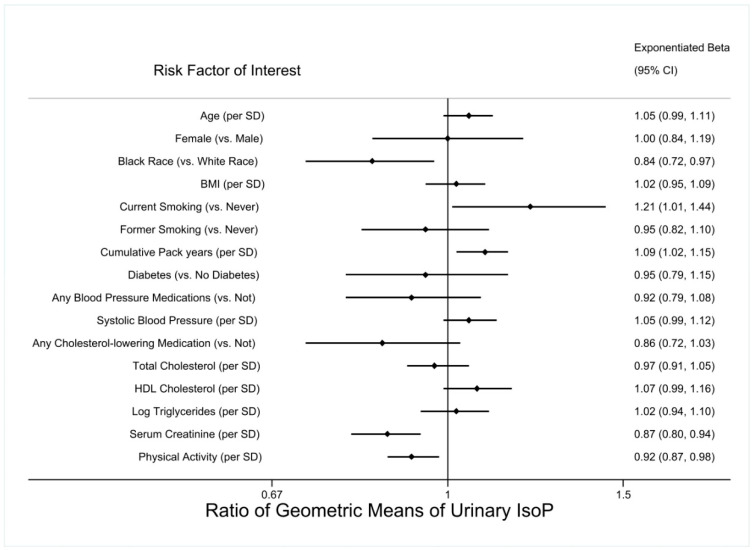
Adjusted associations between IsoP vs. cardiovascular risk factors. Exponentiated beta-coefficients are plotted and are equivalent to geometric ratios of analyte of interest. Ratios > 1 represent positive correlation and ratios < 1 represent inverse correlation. All CVD risk factors are mutually adjusted for one another (age, sex, race, BMI, smoking status, cumulative pack years, diabetes, hypertension medication, systolic blood pressure, lipid-lowering medication, total cholesterol, HDL cholesterol, fasting triglyceride concentrations [log transformed], physical activity) as well as for college attainment and study center. Smoking status and cumulative pack years were highly correlated and thus were not mutually adjusted when one was considered an independent variable of interest.

Mutually adjusted associations between urinary IsoP and CVD risk factors from our primary model are shown in Figure 2. Current smoking (vs. never) and higher cumulative pack years of smoking were associated with higher IsoP. Conversely, more physical activity, higher serum creatinine, and Black race (vs. White) were associated with lower IsoP concentrations. Progressively adjusted associations are reported in Table 2. In model 1, female sex (vs. male) and higher systolic blood pressure (borderline) were also associated with higher IsoP, and cholesterol lowering medications were associated with lower IsoP but their associations were attenuated after further adjustments. HDL-C exhibited borderline significant positive correlation with IsoP after adjustments. No associations between IsoP and age, BMI, diabetes, antihypertensive medication use, triglycerides or total cholesterol were noted regardless of adjustments.

### 3.3. Urinary IsoP m vs. Cardiovascular Risk Factors

**Figure 3:**
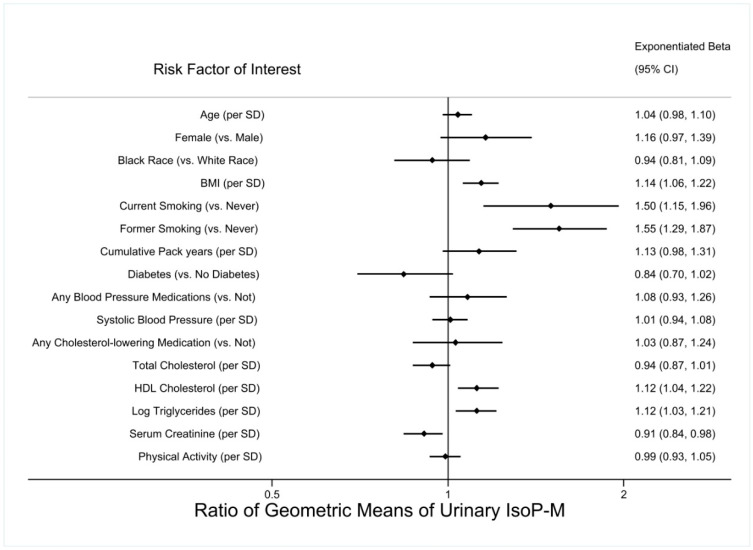
Adjusted associations between IsoP m vs. cardiovascular risk factors. Exponentiated beta-coefficients are plotted and are equivalent to geometric ratios of analyte of interest. Ratios > 1 represent positive correlation and ratios < 1 represent inverse correlation. All CVD risk factors are mutually adjusted for one another (age, sex, race, BMI, smoking status, cumulative pack years, diabetes, hypertension medication, systolic blood pressure, lipid-lowering medication, total cholesterol, HDL cholesterol, fasting triglyceride concentrations [log transformed], physical activity) as well as for college attainment and study center. Smoking status and cumulative pack years were highly correlated and thus were not adjusted for one another in their respective models.

Mutually adjusted associations between urinary IsoP m and CVD risk factors are shown in Figure 3 (progressively adjusted models in Table 3). Similar to IsoP, IsoP m concentrations were higher in female compared with male participants in Model 1, but not in Model 4. However, adjustment for serum creatinine led to only partial attenuation for IsoP m and this association retained its statistical significance with further adjustment for traditional CVD risk factors. Similar to IsoP, current smoking, higher cumulative pack years of smoking and lower serum creatinine were associated with higher IsoP-M. On the other hand, IsoP m exhibited direct relationships with BMI and fasting plasma triglycerides which IsoP had not. IsoP m was not associated with cholesterol-lowering medication use, race, measures of hypertension (systolic pressure or medication use) or physical activity. In the mutually adjusted model, HDL-C was positively associated with IsoP-M- and total cholesterol showed a borderline-significant inverse association, but these associations were not present before adjustments.

### 3.4. Plasma Isoprostanes vs. Cardiovascular Risk Factors

**Figure 4:**
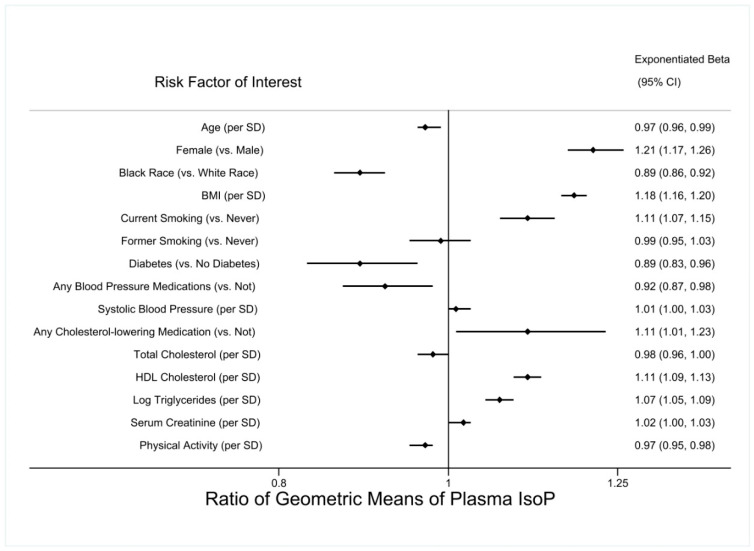
Adjusted associations between Plasma IsoP vs. cardiovascular risk factors. Exponentiated beta-coefficients are plotted and are equivalent to geometric ratios of analyte of interest. Ratios > 1 represent positive correlation and ratios < 1 represent inverse correlation. All CVD risk factors are mutually adjusted for one another (age, sex, race, BMI, smoking status, cumulative pack years, diabetes, hypertension medication, systolic blood pressure, lipid-lowering medication, total cholesterol, HDL cholesterol, fasting triglyceride concentrations [log transformed], physical activity) as well as for college attainment and study center. Smoking status and cumulative pack years were highly correlated and thus were not adjusted for one another in their respective models.

Mutually adjusted associations between plasma IsoP and CVD risk factors are shown in Figure 4 with progressively adjusted models reported in Table 4. Female sex, current smoking, higher BMI, HDL-C, and plasma triglycerides were associated with higher plasma IsoP; however, more physical activity, Black race, and diabetes were associated with lower isoprostane concentrations. Contrary to both urinary assays, plasma IsoP did not demonstrate an inverse association with serum creatinine, and was in fact associated with higher serum creatinine in the main model (model 4).

Some CVD risk factors were associated with plasma IsoP only in specific models. A direct relationship between systolic blood pressure and plasma IsoP was attenuated after adjustments, while hypertension medication use became inversely associated with plasma IsoP in the main model. On the other hand, use of cholesterol-lowering medication was associated with higher plasma IsoP in the final model, but did not show an association in the less adjusted models.

### 3.5. Secondary and Sensitivity Analyses

Findings were similar after adjusting for urinary creatinine as an independent variable instead of indexing (data not shown). Replacing the original variable for diabetes mellitus with HOMA-IR or fasting glucose showed a consistent pattern of lower isoprostanes with impaired glucose metabolism (Supplementary Table S3). Plasma IsoP Models reconstructed in the participant subgroup common to Y15 and Y25 showed no significant change in findings except for the anticipated increase in error estimates (Supplementary Table S4).

In exploratory analysis, we found possible interaction by sex and race between urinary isoprostanes and BMI similar to that which has been reported previously [25,26], such that there was a trend towards an inverse association between BMI and IsoP in men, a trend towards a direct association in women and a trend towards stronger association between IsoP m and BMI in White compared to Black individuals. However, interactions were not statistically significant after Bonferroni correction (Supplementary Table S5 and S6).

## 4. Discussion

Oxidative stress is implicated in the pathogenesis of CVD, and it is regarded as an attractive potential therapeutic and preventive target awaiting a promising breakthrough into clinical practice [27,28,29,30]. However, supporting evidence is largely derived from studies in animals or individuals with advanced CVD, and randomized controlled trials of antioxidant supplementation in at-risk individuals have not shown benefits [31]. Such discordance demands a closer look at oxidative stress in population-level studies. The principal goal of our study was to characterize the relationships between CVD risk factors and oxidative stress in a relatively healthy cohort with appropriate adjustment for potential confounders. We evaluated associations between in vivo oxidative stress as measured by urinary IsoP and its 2,3-dinor metabolite (IsoP-M) with CVD risk factors in a middle-aged subset of a diverse community-based cohort, with additional secondary analysis using previously collected plasma IsoP.

### 4.1. Female Sex

We report higher creatinine-indexed urinary IsoP and IsoP m and higher plasma IsoP in women compared with men in our minimally adjusted models. However, we also found significant confounding by serum creatinine in the urinary assays. Additionally, adjustment for traditional CVD risk factors resulted in further attenuation, such that neither urinary isoprostane showed significant association with sex in the fully adjusted models. Our observations imply higher oxidative stress in women, as measured by isoprostanes; however, the magnitude of this difference may be exaggerated by confounders such as urinary creatinine in urinary assays. Future studies should measure isoprostanes in groups with similar physiology (such as women before and after menopause) and include measurements of sex hormones to more fully investigate potential associations between sex and systemic oxidative stress.

### 4.2. Smoking and Physical Activity

The link between smoking and oxidative stress is well established and it is useful as a positive control for our analysis [16]. After minimal adjustment, all measured isoprostane concentrations were higher in current smokers compared with non-smokers. IsoP m showed >50% higher median among current smokers compared with non-smokers, suggesting this metabolite may be a more discriminative marker for oxidative changes in tobacco users. Interestingly, no clear difference in urine or blood isoprostane concentrations of former smokers (compared with never smokers) was found, implying the reversibility of elevated baseline oxidative stress secondary to tobacco use.

We also found lower baseline oxidative stress in physically active participants, as reflected in lower IsoP in plasma and urine. This pattern was not seen for IsoP-M. As IsoP m is metabolically downstream of IsoP, this could indicate changes in the metabolism of isoprostanes. Studies measuring multiple isoprostane entities and physical activity are scarce, but a similar pattern with IsoP and another metabolite (downstream of IsoP-M) has been reported previously [32], suggesting low physical activity may be associated with IsoP buildup without change in downstream metabolites, albeit clinical significance of this observation remains unknown.

### 4.3. BMI

Higher BMI was associated with higher urinary IsoP m and plasma IsoP, which is consistent with greater oxidative burden in obesity, as reported in previous studies [33], but not with urinary IsoP. The Framingham Heart Study previously reported an association between higher BMI and IsoP measured using enzyme-linked immunosorbent assay (ELISA), especially in women (significant interaction by sex) [25]; however, IsoP m or plasma IsoP were not measured. Insulin Resistance Atherosclerosis Study (IRAS) reported similar findings to our study, in which IsoP measured via GC-MS was not significantly associated with BMI, whereas IsoP m was [34]. While the findings in the IRAS study were not adjusted for other risk factors, our study found this association to be robust after adjustments.

The IRAS study also reported an unexpected inverse relationship between baseline IsoP m and longitudinal weight gain [34]. The authors suggested that production of isoprostanes via lipid oxidation could be a protective compensatory mechanism in response to metabolic changes in obesity. They also found racial differences in associations between BMI and isoprostanes, such that BMI and isoprostane concentrations were correlated in White, but not Black participants, concluding this could signal weaker metabolic adaptability in the latter group [26]. We investigated these previously reported interactions between sex, race and BMI and found a trend toward a sex interaction between BMI and IsoP with non-statistically significant trends towards higher IsoP with higher BMI in women and lower IsoP with higher BMI in men (p-interaction 0.023 in the same direction as the Framingham study did not reach statistical significance after Bonferroni correction). While the interaction beta-coefficients for Black race and BMI were in the same directions reported in the IRAS study (direct association in Whites, no/borderline association in Blacks), this interaction was also not statistically significant (Supplementary Table S5).

### 4.4. Diabetes

Laboratory research suggests that oxidative stress plays an important role in insulin resistance and pancreatic beta cell dysfunction [35,36]. The majority of the few epidemiologic studies assessing isoprostanes in diabetes report a direct cross-sectional relationship between them [16]. However, we found a surprising inverse association between diabetes and plasma IsoP (borderline association in the same direction was also present for IsoP m after adjustments). Replacing our original diabetes variable with other measures of glucose metabolism, such as HOMA-IR and fasting glucose, yielded similar results.

Unexpected associations between isoprostanes and diabetes have been reported before. In one of the early studies, Feillet-Coudray measured higher urinary IsoP but lower plasma IsoP in patients with diabetes compared with controls via ELISA assays [37]. The authors concluded that urinary excretion of isoprostane may be higher in patients with diabetes, though our study shows no associations with urinary isoprostanes. In a more recent study, Ma et al. found no associations between two isoprostane entities measured by GC-MS and insulin resistance, and they suggested the lipid peroxidation process reflected by isoprostanes might be distinct from the oxidative reactions operative in development of insulin resistance [38]. The utility of isoprostanes in prediction of incident diabetes is also contested, and while some reports show higher future risk with high isoprostane concentrations [39], others describe an inverse association [40].

Given the observational and cross-sectional nature of our study, the mechanism underlying our unexpected findings remains unverified. Lower isoprostanes may result from factors that may affect isoprostane production rates, such as changes in lipid metabolism orarachidonic acid availability, changes to esterification (“freeing”) of isoprostanes or effects from certain hypoglycemic agents (we could not account for types of diabetes medications) among other possibilities [17,26]. Future studies that focus on developing our understanding of isoprostane biochemistry, especially in the setting of metabolic abnormalities, are warranted.

### 4.5. Creatinine and Kidney Function

We report a novel, diverging pattern in blood and urinary isoprostane assays. In plasma, higher serum creatinine was associated with higher plasma IsoP after adjustments, which was consistent with higher oxidative stress. Conversely, higher serum creatinine was associated with lower urinary isoprostanes despite exclusion of those with abnormal kidney function, as determined by eGFR. Our study is one of the first reports of the latter paradigm in humans [41] or animals [42] and, to our knowledge, it is the only study to also include plasma isoprostane concentrations.

Urinary isoprostanes are typically indexed to urinary creatinine concentrations to account for variability in urine concentration. Some argue that local production of IsoP in the kidney may limit the generalizability and interpretability of this urinary marker [17,43]. However, if local production was to dominate systemic oxidative stress, it would be expected that higher serum creatinine would correlate with higher urinary isoprostanes, whereas we found an inverse association.

Meanwhile, a rarely considered issue is the dependency of urinary creatinine concentrations not only on the kidneys’ ability to filter and concentrate urine, but also its original concentration in the serum. Differences in serum creatinine (which correlate with body size, age and sex) could result in differences in urine creatinine irrespective of renal function and hydration, and thus, factors associated with differences in serum creatinine could confound associations of urinary creatinine indexed isoprostanes. In our analyses, we included serum creatinine as an early adjustment factor and also excluded individuals with eGFR <60 mg/dL/m^2^ to account for these confounders, and we found this to be particularly relevant in assessment of sex differences in oxidative stress. Our findings highlight the importance of adjustment for serum creatinine as a confounder in all future analyses of urinary isoprostanes, particularly if groups of interest are expected to have different serum concentrations.

### 4.6. Hypertension

In our minimally adjusted models, IsoP concentrations were higher in urine and plasma in participants with higher systolic blood pressure, while use of antihypertensive medication was associated with lower plasma IsoP. This could be consistent with higher oxidative stress in those with uncontrolled hypertension [44] and reduction of oxidative stress achieved using commonly used antihypertensive medications [45]. However, given the nature of our study, we are unable to distinguish whether these differences stem from the vascular endothelium itself, secondary to end-organ distress, or are not causal at all. Future studies should consider blood pressure and antihypertensive use as independent factors that could affect isoprostane concentrations and use adjustments accordingly.

### 4.7. Plasma Lipids and Cholesterol Medications

Statins, the cornerstone in lipid-lowering therapy, are reported to have pleiotropic effects ranging from lowering low-density lipoprotein cholesterol to reducing inflammation, and they are also believed to reduce oxidative stress [46,47]. On the other hand, reports to the contrary also exist [48,49]. Moreover, some studies suggest within-class differences [50,51,52], or similar effects from other types of cholesterol-lowering therapies [53].

In our study, cholesterol-lowering medication use was associated with lower IsoP, not associated with IsoP-M and associated with higher plasma IsoP (in some models). It is important to consider that isoprostanes are the result of lipid peroxidation themselves. Hence, associations may be due to changes in the metabolism and excretion of isoprostanes as opposed to true differences in in vivo oxidative stress [17]. Alternatively, the participant groups using these medications may not be entirely comparable in our urinary isoprostane vs. plasma isoprostane analysis due to differences in the timing of isoprostane sampling. At the time of plasma IsoP measurements, CARDIA participants were roughly 40 years old (Supplementary Table S2), so cholesterol-lowering pharmacotherapy in this group could be indicative of early dyslipidemia, or alternatively earlier access to outpatient medical care. Urinary isoprostanes were measured 10 years later when the average participant age was >52 years old, an age at which initiation of statin therapy is considerably more common.

We report direct associations between higher fasting triglycerides and isoprostanes in urine IsoP m and blood IsoP. Similar associations have been previously reported, but as secondary findings and without adjustment for other CVD risk factors such as BMI [20,54]. Our results may suggest higher oxidative stress in hypertriglyceridemia, which can contribute to atherosclerotic disease.

Contrary to triglycerides, HDL lipoproteins are often touted as a “scavenger” of oxidized lipids and are considered cardioprotective unless in extremely high concentrations [55]. In vitro studies have shown HDL (HDL_3_ in particular) to be the main lipoprotein carrier of isoprostanes in the blood [56], which may be consistent with their involvement in removal of these byproducts of oxidative stress. As urinary isoprostane assays measure de-esterified and hydrolyzed isoprostanes “freed” from the cells [17], a direct association linking higher HDL concentration and higher isoprostanes may be linked to increased “freeing” of this entity, resulting in higher measured plasma or urine concerntrations, despite potentially lower oxidative stress in tissues. Other potential mechanisms for this association may be differences in the quality of measured HDL, such as particle subtypes or sizes. The exact pathophysiologic mechanisms linking HDL cholesterol, isoprostanes and CVD remain unknown, and whether this association is cardioprotective or not requires further investigation.

### 4.8. Strengths and Limitations

There are limitations to the presented study. Isoprostanes were measured only once per participant, and therefore we are unable to assess potential chronologic variations or longitudinal associations between oxidative stress and development of CVD risk factors. There are several methods used for isoprostane measurement, and the field lacks general assay standardization, limiting the interpretability of findings across studies. We used GC-MS, which is often considered the gold standard [57], though measurement inaccuracies are unavoidable. Due to the observational nature of the study, we cannot differentiate the effects of some risk factors (such as diabetes) from the effects of medications used to treat them, which may affect isoprostane concentrations or oxidative stress in general. We were bound by the inclusion/exclusion criteria that suited the original ancillary study, which included measurement of urinary isoprostanes, even though some of these criteria do not apply directly to the goals of our study. Our secondary analysis used plasma isoprostane measurements predating our primary urinary isoprostane analysis by 10 years and measuring total F2-isoprostanes without distinction between 8-isoprostanes and other subtypes. Therefore, comparison of associations may be limited by differences in prevalence of CVD risk factors present at each timepoint, differences in numbers of participants and the fact that isoprostane subtypes may have different association patterns (though demonstration of this point is arguably a strength of our analysis).

Our study also has many strengths, including analysis of both IsoP and its metabolite IsoP m, which would be impervious to previously cited concerns about local production in the kidney and could add to the limited body of literature about differences in associations between risk factors and isoprostane sub-classes. Our results are further strengthened by inclusion of both urinary and plasma isoprostanes which, to our knowledge, is occurring for the first time in a community cohort study. Use of urinary isoprostanes as our primary biomarker is robust against ex vivo production of isoprostanes which would otherwise exhibit a great challenge in the previously collected samples typically available in biobanks of observational cohort studies. Our study population was derived from a well-characterized cohort, which allowed for adjustment for numerous potential factors as well as investigation of many CVD risk factors using standardized protocols. In addition, our study was performed in a relatively young and healthy population, while the majority of existing studies focus on populations with advanced disease.

## 5. Conclusions

We provide broad and detailed analysis of associations between traditional risk factors for CVD, with urinary and plasma isoprostane concentrations which are well regarded as qualitative measures of in vivo systemic oxidative stress. Assays used in our study exhibited varying degrees of discriminatory power (and, in some cases, opposite correlations) with specific CVD risk factors, such that both the type of body fluid analyzed and the isoprostane entity measured could affect the observed associations. As such, it is best to consider isoprostanes as complementary, rather than exchangeable, in snapshot assessments of in vivo oxidative stress. In addition, while urinary isoprostanes are convenient, indexing to urine creatinine could result in significant confounding when groups with physiologically different serum creatinine concentrations are compared. Still, consistently higher isoprostane concentrations observed among women, smokers, sedentary persons and in those with higher BMI and plasma triglycerides could reflect higher oxidative stress, contributing to greater CVD risk in those groups.

## Figures and Tables

**Table 1:** Characteristics of participants at CARDIA Year 25 exam.

	Women (*n* = 475)	Men (*n* = 266)	*p*
**Age, years**	52.2 ± 2.2	52.1 ± 2.3	0.46
**Postmenopausal**	279 (58.7%)	-	-
**Black Race**	183 (38.5%)	106 (39.8%)	0.72
**Body Mass Index (kg/m^2^)**	30.0 ± 7.8	29.0 ± 5.8	0.06
**College Attainment**	280 (59.1%)	139 (52.3%)	0.07
**Smoking**			0.68
**Never**	288 (61.0%)	169 (63.8%)	
**Former**	118 (25.3%)	59 (22.3%)	
**Current**	63 (13.6%)	37 (13.8%)	
**Cumulative Pack years**	1.0 ± 3.4	1.6 ± 5.0	0.06
Physical Activity, EU *	304 ± 230	404 ± 286	**<0.01**
**Systolic Blood Pressure, mmHg**	116 ± 16	121 ± 15	**<0.01**
**Total Cholesterol, mg/dL**	198 ± 36	189 ± 35	**<0.01**
**HDL Cholesterol, mg/dL**	65 ± 17	51 ± 16	**<0.01**
**Triglycerides, mg/dL**	98 ± 59	136 ± 138	**<0.01**
**Serum Creatinine, mg/dL**	0.75 ± 0.12	0.97 ± 0.16	**<0.01**
**Diabetes**	54 (11.4%)	47 (17.7%)	**0.02**
**Fasting Glucose, mg/dL**	95 ± 21	105 ± 31	**<0.01**
**Antihypertensive Medication Use**	101 (21.2%)	76 (28.6%)	**0.03**
**Cholesterol Lowering Medication Use**	62 (13.2%)	49 (18.6%)	**0.05**
**Urinary Creatinine, mg/dL**	105 ± 72	150 ± 86	**<0.01**
**Urinary IsoP, ng/g Creatinine ^†^**	880 [75, 1379]	704 [464, 1019]	**<0.01**
**Urinary IsoP-M, ng/g Creatinine ^‡^**	1675 [1118, 2726]	1284 [788, 2037]	**<0.01**

Data presented as mean ± SD, median [IQR], or percentage of participants. Statistically significant results (*p* < 0.05) **bold**ed. * EU: Exercise units **^†^ IsoP**: 8-isoprostane **^‡^ IsoP-M**: 2,3-dinor-8-isoprostane.

**Table 2:** Adjusted associations between IsoP vs. cardiovascular risk factors.

	Model 1 (*n* = 738)	Model 2 (*n* = 738)	Model 3 (*n* = 731)	Model 4 (*n* = 723)
**Age (per SD)**	1.04 (0.98 to 1.10)	1.05 (0.99 to 1.11)	1.05 (0.99 to 1.12)	1.05 (0.99 to 1.11)
**Female Sex (vs. Male)**	**1.28** **(1.14 to 1.45)**	1.04 (0.89 to 1.22)	1.07 (0.91 to 1.25)	1.00 (0.84 to 1.19)
**Black Race (vs. White)**	**0.83** **(0.72 to 0.94)**	0.88 (0.77 to 1.01)	0.87 (0.76 to 1.00)	**0.84** **(0.72 to 0.97)**
**Body Mass Index (per SD)**	1.01 (0.95 to 1.07)	1.00 (0.95 to 1.07)	1.00 (0.94 to 1.07)	1.02 (0.95 to 1.09)
**Current Smoking** (vs. Never) *	**1.23** **(1.03 to 1.47)**	**1.20** **(1.01 to 1.43)**	-	**1.21** **(1.01 to 1.44)**
**Former Smoking** (vs. Never) *	0.93 (0.81 to 1.08)	0.94 (0.82 to 1.09)	-	0.95 (0.82 to 1.10)
**Cumulative Pack years** (per SD) *	**1.10** **(1.04 to 1.17)**	**1.09** **(1.03 to 1.15)**	-	**1.09** **(1.02 to 1.15)**
**Diabetes**	0.94 (0.79 to 1.12)	0.91 (0.77 to 1.09)	0.90 (0.75 to 1.07)	0.95 (0.79 to 1.15)
**Blood Pressure Medication**	0.92 (0.80 to 1.07)	0.92 (0.80 to 1.07)	0.91 (0.79 to 1.05)	0.92 (0.79 to 1.08)
**Systolic BP (per SD)**	1.06 (1.00 to 1.13)	1.05 (0.98 to 1.12)	1.05 (0.98 to 1.12)	1.05 (0.99 to 1.12)
**Cholesterol-lowering** **Medication**	**0.84** **(0.71 to 0.99)**	**0.84** **(0.71 to 0.99)**	**0.84** **(0.72 to 1.00)**	0.86 (0.72 to 1.03)
**Total Cholesterol (per SD)**	1.01 (0.95 to 1.07)	1.02 (0.96 to 1.08)	1.02 (0.96 to 1.08)	0.97 (0.91 to 1.05)
**HDL Cholesterol (per SD)**	1.05 (0.98 to 1.12)	1.05 (0.98 to 1.12)	1.06 (0.99 to 1.13)	1.07 (0.99 to 1.16)
**Log Triglycerides (per SD)**	1.00 (0.94 to 1.06)	1.00 (0.94 to 1.07)	0.99 (0.93 to 1.06)	1.02 (0.94 to 1.10)
**Serum Creatinine (per SD)**	**0.85** **(0.79 to 0.92)**	-	**0.86** **(0.80 to 0.93)**	**0.87** **(0.80 to 0.94)**
**Physical Activity (per SD)**	**0.92** **(0.87 to 0.98)**	**0.92** **(0.87 to 0.98)**	**0.93** **(0.87 to 0.98)**	**0.92** **(0.87 to 0.98)**

Statistically significant results (*p* < 0.05) were **bold**ed. Exponentiated beta-coefficients are equivalent to geometric ratios of analyte of interest. Ratios > 1 represent positive correlation and ratios < 1 represent inverse correlation. Models were progressively adjusted as follows: Model 1—Single CV risk factor + age, sex, race, BMI, college attainment, study center; Model 2—Single CV risk factor + Model 1 +serum creatinine; Model 3 *—Single CV risk factor + Model 2 + smoking status (current, former, or never) + cumulative pack years; Model 4—All CV risk factors mutually adjusted (diabetes, hypertension medication, systolic blood pressure, lipid-lowering medication, total cholesterol, HDL cholesterol, fasting triglyceride concentrations [log transformed], physical activity) + Model 3; * Smoking status and cumulative pack years were highly correlated and thus were not adjusted for one another in their respective models.

**Table 3:** Adjusted associations between IsoP m vs. cardiovascular risk factors.

	Model 1 (*n* = 738)	Model 2 (*n* = 738)	Model 3 (*n* = 731)	Model 4 (*n* = 723)
**Age (per SD)**	1.04 (0.98 to 1.10)	1.05 (0.99 to 1.11)	1.05 (0.99 to 1.11)	1.04 (0.98 to 1.10)
**Female Sex (vs. Male Sex)**	**1.36** **(1.20 to 1.54)**	**1.18** **(1.01 to 1.38)**	**1.20** **(1.02 to 1.40)**	1.16 (0.97 to 1.39)
**Black** **Race (vs. White)**	0.92 (0.80 to 1.05)	0.96 (0.83 to 1.10)	0.94 (0.82 to 1.08)	0.94 (0.81 to 1.09)
**BMI (per SD)**	**1.13** **(1.06 to 1.20)**	**1.13** **(1.06 to 1.20)**	**1.13** **(1.06 to 1.20)**	**1.14** **(1.06 to 1.22)**
**Current Smoking** (vs. Never)*	**1.61** **(1.35 to 1.93)**	**1.59** **(1.33 to 1.90)**	-	**1.55** **(1.22 to 1.87)**
**Former Smoking** (vs. Never)*	1.14 (0.99 to 1.31)	1.15 (0.99 to 1.32)	-	1.13 (0.98 to 1.31)
**Cumulative Pack years** (per SD)*	**1.13** **(1.07 to 1.20)**	**1.13** **(1.06 to 1.20)**	-	**1.12** **(1.06 to 1.19)**
**Diabetes**	0.88 (0.72 to 1.08)	0.86 (0.70 to 1.06)	0.86 (0.70 to 1.06)	0.84 (0.70 to 1.02)
**Blood Pressure Medication**	1.10 (0.95 to 1.28)	1.10 (0.96 to 1.28)	1.09 (0.95 to 1.26)	1.08 (0.93 to 1.26)
**Systolic BP (per SD)**	1.04 (0.97 to 1.10)	1.03 (0.96 to 1.09)	1.02 (0.96 to 1.09)	1.01 (0.95 to 1.08)
**Cholesterol-lowering Meds**	1.06 (0.89 to 1.25)	1.06 (0.90 to 1.25)	1.05 (0.89 to 1.24)	1.03 (0.87 to 1.24)
**Total Cholesterol (per SD)**	1.00 (0.94 to 1.06)	1.01 (0.95 to 1.07)	1.00 (0.94 to 1.06)	0.94 (0.87 to 1.01)
**HDL Cholesterol (per SD)**	1.06 (0.99 to 1.14)	1.06 (0.99 to 1.13)	1.06 (0.99 to 1.13)	**1.12** **(1.04 to 1.22)**
**Log Triglycerides (per SD)**	**1.07** **(1.00 to 1.14)**	**1.07** **(1.01 to 1.14)**	1.05 (0.99 to 1.12)	**1.12** **(1.03 to 1.21)**
**Serum Creatinine (per SD)**	**0.90** **(0.83 to 0.97)**	**0.90** **(0.83 to 0.97)**	**0.91** **(0.84 to 0.98)**	**0.91** **(0.84 to 0.98)**
**Physical Activity (per SD)**	0.98 (0.92 to 1.04)	0.98 (0.92 to 1.04)	0.98 (0.93 to 1.05)	0.99 (0.93 to 1.05)

Statistically significant results (*p* < 0.05) were **bold**ed. Exponentiated beta-coefficients are equivalent to geometric ratios of analyte of interest. Ratios >1 represent positive correlation and ratios <1 represent inverse correlation. Models were progressively adjusted as follows: Model 1—Single CV risk factor + age, sex, race, BMI, college attainment, study center; Model 2—Single CV risk factor + Model 1 +serum creatinine; Model 3 *—Single CV risk factor + Model 2 + smoking status (current, former, or never) + cumulative pack years; Model 4—All CV risk factors mutually adjusted (diabetes, hypertension medication, systolic blood pressure, lipid-lowering medication, total cholesterol, HDL cholesterol, fasting triglyceride concentrations [log transformed], physical activity) + Model 3; * Smoking status and cumulative pack years were highly correlated and thus were not adjusted for one another in their respective models.

**Table 4:** Adjusted associations between Plasma IsoP vs. cardiovascular risk factors.

	Model 1	Model 2	Model 3	Model 4
**Age (per SD)**	**0.98** **(0.97 to 1.00)**	**0.98** **(0.97 to 1.00)**	**0.98** **(0.97 to 1.00)**	**0.97** **(0.96 to 0.99)**
**Female Sex (vs. Male Sex)**	**1.26** **(1.23 to 1.30)**	**1.27** **(1.23 to 1.31)**	**1.28** **(1.24 to 1.32)**	**1.21** **(1.17 to 1.26)**
**Black** **Race (vs. White)**	**0.90** **(0.87 to 0.93)**	**0.89** **(0.86 to 0.92)**	**0.89** **(0.86 to 0.92)**	**0.89** **(0.86 to 0.92)**
**BMI (per SD)**	**1.16** **(1.14 to 1.18)**	**1.16** **(1.14 to 1.18)**	**1.17** **(1.15 to 1.18)**	**1.18** **(1.16 to 1.20)**
**Current Smoking** **(vs. Never)**	**1.12** **(1.08 to 1.16)**	**1.12** **(1.08 to 1.16)**	**1.12** **(1.08 to 1.16)**	**1.11** **(1.07 to 1.15)**
**Former Smoking (vs. Never)**	1.00 (0.96 to 1.04)	1.00 (0.96 to 1.04)	1.00 (0.96 to 1.04)	0.99 (0.95 to 1.03)
Cumulative Pack years*	Not Available			
**Diabetes**	**0.90** **(0.84 to 0.93)**	**0.90** **(0.83 to 0.97)**	**0.89** **(0.83 to 0.96)**	**0.89** **(0.83 to 0.96)**
**Blood Pressure Medication**	0.95 (0.90 to 1.00)	**0.94** **(0.89 to 1.00)**	0.95 (0.89 to 1.00)	**0.92** **(0.87 to 0.98)**
**Systolic BP (per SD)**	**1.02** **(1.00 to 1.04)**	1.02 (1.00 to 1.03)	1.02 (1.00 to 1.03)	1.01 (1.00 to 1.03)
**Cholesterol-lowering Meds**	1.07 (0.97 to 1.18)	1.07 (0.97 to 1.18)	1.07 (0.97 to 1.19)	1.11 (1.01 to 1.23)
**Total Cholesterol (per SD)**	**1.02** **(1.00 to 1.03)**	**1.02** **(1.00 to 1.03)**	**1.02** **(1.00 to 1.03)**	**0.98** **(0.96 to 1.00)**
**HDL Cholesterol (per SD)**	**1.07** **(1.05 to 1.09)**	**1.07** **(1.05 to 1.09)**	**1.07** **(1.06 to 1.09)**	**1.11** **(1.09 to 1.13)**
**Log Triglycerides (per SD)**	**1.03** **(1.01 to 1.04)**	**1.03** **(1.01 to 1.05)**	**1.02** **(1.01 to 1.04)**	**1.07** **(1.05 to 1.10)**
**Serum Creatinine (per SD)**	1.01 (1.00 to 1.03)	1.01 (1.00 to 1.03)	1.01 (1.00 to 1.03)	**1.02** **(1.00 to 1.03)**
**Physical Activity (per SD)**	**0.97** **(0.96 to 0.99)**	**0.97** **(0.96 to 0.99)**	**0.98** **(0.96 to 0.99)**	**0.97** **(0.95 to 0.98)**

Statistically significant results (*p* < 0.05) were **bold**ed. Exponentiated beta-coefficients are equivalent to geometric ratios of analyte of interest. Ratios >1 represent positive correlation and ratios < 1 represent inverse correlation. Models were progressively adjusted as follows: Model 1—Single CV risk factor + age, sex, race, BMI, college attainment, study center; Model 2—Single CV risk factor + Model 1 +serum creatinine; Model 3 *—Single CV risk factor + Model 2 + smoking status (current, former, or never); Model 4—All CV risk factors mutually adjusted (diabetes, hypertension medication, systolic blood pressure, lipid-lowering medication, total cholesterol, HDL cholesterol, fasting triglyceride concentrations [log transformed], physical activity) + Model 3; * Cumulative pack years was not available for the Y15 visit.

## Data Availability

The data presented in this study are available on request from the CARDIA cohort.

## Supplementary Materials

The following supporting information can be downloaded at: https://www.mdpi.com/article/10.3390/antiox12030555/s1, Table S1: Quality control metrics of urinary assays; Table S2: Characteristics of participants at CARDIA Y15 exam; Table S3: Replacing different diabetes-related variables into the models; Table S4: Plasma Iso-P Models in Participants and in Urine Isoprostane Analyses; Table S5: *p*-values for interaction terms between each of the covariates and sex or race in the fully adjusted model; Table S6: Interactions between Sex/Race and BMI on urinary isoprostanes.
