# Supplementary material for: Oxidative Stress and Cardiovascular Risk Factors: The Coronary Artery Risk Development in Young Adults (CARDIA) Study
Source: Antioxidants (Basel). 2023 Feb 23;12(3):555. doi: 10.3390/antiox12030555 (PMC10044794; doi:10.3390/antiox12030555)
Supplement: Supplementary file 1 [file antioxidants-12-00555-s001.zip › antioxidants-2168325-supplementary.pdf]

Supplements

Supplementary Table S1. Quality control metrics of urinary assays

	IsoP		IsoP-M	
	**Control Set 1**	**Control Set 2**	**Control Set 1**	**Control Set 2**
**Number of Sample Runs, *n***	56	50	50	50
**Concentration (mean ± SD), ng/ml**	4.1 ± 0.9	17.8 ± 3.9	3.6 ± 0.9	13.7 ± 3.1
**Intra-assay CV***	6.7%	5.2%	6.5%	6.5%
**Inter-assay CV***	21.7%	21.7%	24.1%	22.9%

For each isoprostane entity, 2 pools of quality control samples with low and high concentration were included as duplicates in each run/batch. For each run, standard deviation of each duplicated sample divided by duplicate mean yielded sample specific intra-assay CV. Mean sample specific intraassay CV for each control set is reported as pool intra-assay CV. Sample specific Inter-assay CV was calculated as the standard deviation for mean concentration of each sample across the batches/runs divided by mean for that sample across the batches. Control set inter-assay CV was then calculated as mean of sample specific inter-assay CVs in each pool.
* CV: Coefficient of variance

Supplementary Table S2. Characteristics of participants at CARDIA Y15 exam

	Female (*n*= 1623)	Male (*n*= 1376)	*p*
**Age, years**	40.3 ± 3.6	40.2 ± 3.6	0.40
**Postmenopausal**	12.1%	-	-
**Black Race**	47.3%	42.1%	<0.01
**Body Mass Index (BMI; kg/m^2)**	28.9 ± 7.2	28.0 ± 4.9	<0.01
**≥ College degree**	48.5%	45.6%	0.15
**Smoking**			
**Never**	59.8%	61.8%	<0.01
**Former**	20.8%	16.0%	
**Current**	19.4%	22.2%	
**Cumulative Pack-Years**	Not Available	Not available	-
**Physical Activity, EU ***	290 ± 249	429 ± 307	<0.01
**Systolic Blood Pressure, mmHg**	111 ± 15	115 ± 14	<0.01
**Total Cholesterol, mg/dl**	182 ± 32	189 ± 38	<0.01
**HDL Cholesterol, mg/dl**	55 ± 14	45 ± 13	<0.01
**Triglycerides, mg/dl**	89 ± 58	126 ± 124	<0.01
**Serum Creatinine, mg/dl**	0.76 ± 0.24	1.00 ± 0.50	<0.01
**Diabetes Medication Use**	2.3%	2.0%	0.65
**Fasting Glucose, mg/dl**	92 ± 20	96 ± 19	<0.01
**Antihypertensive Medication Use**	7.4%	7.3%	0.95
**Cholesterol Lowering Medication Use**	3.9%	1.0%	<0.01
**Plasma IsoP, ng/L†**	57.2 [41.8, 80.9]	45.9 [36.9, 59.5]	<0.01

Data presented as mean ± SD, median [IQR], or percentage of participants
*** EU:** Exercise units **† IsoP:** 8-isoprostane

Supplementary Table S3. Replacing different diabetes-related variables into the models

	Diabetes-related variable	Model 1	Model 2	Model 3	Model 4
Urinary IsoP	**Original DM variable***	0.94 (0.79 to 1.12)	0.91 (0.77 to 1.09)	0.90 (0.75 to 1.07)	0.95 (0.79 to 1.15)
	**Hemoglobin A1c (per SD)**	0.99 (0.93 to 1.05)	0.98 (0.92 to 1.04)	0.97 (0.91 to 1.03)	1.00 (0.92 to 1.07)
	**Log HOMA-IR (per SD)**	0.96 (0.90 to 1.04)	0.96 (0.89 to 1.03)	0.96 (0.90 to 1.04)	0.96 (0.88 to 1.04)
	**Fasting Glucose (per SD)**	1.01 (0.95 to 1.07)	1.00 (0.94 to 1.06)	0.99 (0.93 to 1.05)	1.01 (0.94 to 1.07)
	**Diabetes Medication use**	1.04 (0.83 to 1.30)	1.01 (0.81 to 1.27)	1.00 (0.80 to 1.25)	1.14 (0.88 to 1.46)
Urinary IsoP-M	**Original DM variable***	0.88 (0.72 to 1.08)	0.86 (0.70 to 1.06)	0.86 (0.70 to 1.06)	**0.84 (0.70 to 1.02)**
	**Hemoglobin A1c (per SD)**	0.99 (0.93 to 1.06)	0.99 (0.93 to 1.05)	0.98 (0.92 to 1.04)	1.00 (0.93 to 1.08)
	**Log HOMA-IR (per SD)**	0.94 (0.88 to 1.01)	0.94 (0.87 to 1.01)	0.95 (0.88 to 1.02)	**0.91 (0.84 to 0.99)**
	**Fasting Glucose (per SD)**	1.00 (0.94 to 1.06)	0.99 (0.93 to 1.05)	0.98 (0.92 to 1.04)	0.97 (0.91 to 1.03)
	**Diabetes Medication use**	1.01 (0.81 to 1.27)	1.00 (0.79 to 1.25)	0.99 (0.79 to 1.24)	0.91 (0.71 to 1.17)
Plasma IsoP	**Original DM variable***	**0.90 (0.84 to 0.93)**	**0.90 (0.83 to 0.97)**	**0.89 (0.83 to 0.96)**	**0.89 (0.83 to 0.96)**
	**Hemoglobin A1c (per SD)**	-	-	-	-
	**Log HOMA-IR (per SD)**	0.99 (0.97 to 1.00)	0.99 (0.97 to 1.00)	0.98 (0.97 to 1.00)	**0.98 (0.96 to 1.00)**
	**Fasting Glucose (per SD)**	**0.97 (0.95 to 0.98)**	**0.97 (0.95 to 0.98)**	**0.97 (0.95 to 0.98)**	**0.96 (0.95 to 0.98)**
	**Diabetes Medication use**	**0.87 (0.79 to 0.96)**	**0.86 (0.78 to 0.95)**	**0.86 (0.78 to 0.95)**	**0.85 (0.77 to 0.94)**

Models were constructed as described in the methods section, with each of the listed diabetes-related variables being replaced in each model separately to assess robustness of the associations to variable definition.

*** Diabetes:** use of diabetes medications or fasting glucose ≥126 mg/dL

Supplementary Table S4. Plasma Iso-P Models in Participants Also in Urine Isoprostane Analyses

	**Model 1**		**Model 4**	
	All Y15 Participants	Overlapping Participants	All Y15 Participants	Overlapping Participants
**Age (per SD)**	**0.98 (0.97 to 1.00)**	1.00 (0.97 to 1.04)	**0.97 (0.96 to 0.99)**	0.99 (0.96 to 1.02)
**Female Sex (vs. Male Sex)**	**1.26 (1.23 to 1.30)**	**1.24 (1.16 to 1.32)**	**1.21 (1.17 to 1.26)**	**1.27 (1.15 to 1.40)**
**Black Race (vs. White)**	**0.90 (0.87 to 0.93)**	**0.86 (0.80 to 0.93)**	**0.89 (0.86 to 0.92)**	**0.86 (0.79 to 0.93)**
**BMI (per SD)**	**1.16 (1.14 to 1.18)**	**1.18 (1.14 to 1.23)**	**1.18 (1.16 to 1.20)**	**1.18 (1.13 to 1.22)**
**Current Smoking (vs. Never)**	**1.12 (1.08 to 1.16)**	**1.10 (1.00 to 1.20)**	**1.11 (1.07 to 1.15)**	1.09 (0.99 to 1.19)
**Diabetes***	**0.90 (0.84 to 0.93)**	**0.94 (0.79 to 1.11)**	**0.89 (0.83 to 0.96)**	0.95 (0.81 to 1.12)
**Blood Pressure Medication**	0.95 (0.90 to 1.00)	0.93 (0.82 to 1.07)	**0.92 (0.87 to 0.98)**	**0.83 (0.72 to 0.95)**
**Systolic BP (per SD)**	**1.02 (1.00 to 1.04)**	**1.05 (1.02 to 1.09)**	1.01 (1.00 to 1.03)	**1.05 (1.02 to 1.09)**
**Cholesterol-lowering Meds**	1.07 (0.97 to 1.18)	1.07 (0.82 to 1.39)	**1.11 (1.01 to 1.23)**	**1.14 (0.88 to 1.47)**
**Total Cholesterol (per SD)**	**1.02 (1.00 to 1.03)**	1.01 (0.98 to 1.05)	**0.98 (0.96 to 1.00)**	**0.95 (0.92 to 0.99)**
**HDL Cholesterol (per SD)**	**1.07 (1.05 to 1.09)**	**1.06 (1.02 to 1.10)**	**1.11 (1.09 to 1.13)**	**1.12 (1.07 to 1.17)**
**Log Triglycerides (per SD)**	**1.03 (1.01 to 1.04)**	**1.06 (1.02 to 1.10)**	**1.07 (1.05 to 1.10)**	**1.12 (1.07 to 1.16)**
**Serum Creatinine (per SD)**	1.01 (1.00 to 1.03)	1.02 (0.98 to 1.07)	**1.02 (1.00 to 1.03)**	1.04 (1.00 to 1.09)
**Physical Activity (per SD)**	**0.97 (0.96 to 0.99)**	0.97 (0.94 to 1.00)	**0.97 (0.95 to 0.98)**	**0.96 (0.92 to 0.99)**

Statistically significant results (p<0.05) were **bold**ed. Exponentiated beta-coefficients are equivalent to geometric ratios of analyte of interest. Ratios >1 represent positive correlation and ratios <1 represent inverse correlation.
Models were progressively adjusted as follows:
Model 1—Single CV risk factor + age, sex, race, BMI, college attainment, study center;
Model 4—All CV risk factors mutually adjusted (diabetes, hypertension medication, systolic blood pressure, lipid lowering medication, total cholesterol, HDL cholesterol, fasting triglyceride concentrations [log transformed], physical activity) +serum creatinine + smoking status (current, former, or never) + Model 1;
*** Diabetes:** use of diabetes medications or fasting glucose ≥126 mg/dL

Supplementary Table S5. P values for interaction terms between each of the covariates and sex or race in the fully adjusted model

	P value for Interaction term			
	Sex		Race	
	IsoP	IsoP-M	IsoP	IsoP-M
**Black Race (vs. White)**	0.42	0.34	-	-
**BMI (per SD)**	0.02	0.28	0.07	0.05
**Current Smoking (vs. Never)**	0.42	0.71	0.24	0.58
**Former Smoking (vs. Never)**	0.03	0.50	0.84	0.48
**Cumulative Pack-years**	0.77	0.71	0.50	0.60
**Diabetes**	0.54	0.82	0.14	0.75
**Blood Pressure Medication**	0.99	0.49	0.07	0.12
**Systolic BP (per SD)**	0.26	0.29	0.25	0.02
**Cholesterol-lowering Meds**	0.17	0.83	0.07	0.02
**Total Cholesterol (per SD)**	0.06	0.11	0.17	0.27
**HDL Cholesterol (per SD)**	0.57	0.81	0.75	0.58
**Log Triglycerides (per SD)**	0.27	0.01	0.81	0.16
**Serum Creatinine (per SD)**	0.04	0.40	0.95	0.97
**Physical Activity (per SD)**	0.21	0.89	0.02	0.46

Supplementary Table S6. Interactions between Sex/Race and BMI on urinary isoprostanes

	β for Male	β for Female	*p*	β for Black	β for White	*p*
**IsoP**	0.89 (0.79-1.02)	1.05 (0.98-1.14)	0.02	1.09 (0.98-1.20)	0.97 (0.89-1.06)	0.07
**IsoP-M**	1.07 (0.94-1.22)	1.16 (1.07-1.25)	0.28	1.22 (1.11-1.35)	1.08 (0.99-1.18)	0.05
